# Expression of Cytokines and Neurodegeneration in the Rat Hippocampus and Cortex in the Lithium-Pilocarpine Model of Status Epilepticus and the Role of Modulation of Endocannabinoid System

**DOI:** 10.3390/ijms24076509

**Published:** 2023-03-30

**Authors:** Elena M. Suleymanova, Anna A. Karan, Maria A. Borisova, Maria N. Volobueva, Alexey P. Bolshakov

**Affiliations:** Institute of Higher Nervous Activity and Neurophysiology of the Russian Academy of Sciences, Butlerova Street 5A, 117485 Moscow, Russia

**Keywords:** neuroinflammation, cytokines, status epilepticus, endocannabinoid receptors, neurodegeneration, epilepsy

## Abstract

A significant body of evidence shows that neuroinflammation is one of the key processes in the development of brain pathology in trauma, neurodegenerative disorders, and epilepsy. Various brain insults, including severe and prolonged seizure activity during status epilepticus (SE), trigger proinflammatory cytokine release. We investigated the expression of the proinflammatory cytokines interleukin-1β (*Il1b*) and interleukin-6 (*Il6*), and anti-inflammatory fractalkine (*Cx3cl1*) in the hippocampus, entorhinal cortex, and neocortex of rats 24 h, 7 days, and 5 months after lithium-pilocarpine SE. We studied the relationship between cytokine expression and neuronal death in the hippocampus and evaluated the effect of modulation of endocannabinoid receptors on neuroinflammation and neurodegeneration after SE. The results of the present study showed that inhibition of endocannabinoid CB1 receptors with AM251 early after SE had a transient neuroprotective effect that was absent in the chronic period and did not affect the development of spontaneous seizures after SE. At the same time, AM251 reduced the expression of Il6 in the chronic period after SE. Higher *Cx3cl1* levels were found in rats with more prominent hippocampal neurodegeneration.

## 1. Introduction

A large body of evidence has been accumulated for the involvement of neuroinflammation in many pathological processes in the brain. In particular, many studies clearly show that inflammation is involved in the pathogenesis of epilepsy [1], which is one of the most common neurological disorders affecting large populations of people across the world. Neuroinflammation is involved in the mechanisms underlying increased excitability and epileptogenesis [1,2]. Microglia and astrocytes are rapidly activated by various epileptic stimuli and begin to synthesize and secrete a variety of neurotrophic and neurotoxic factors, including proinflammatory and anti-inflammatory cytokines, many of which have a neuromodulatory function [3]. Activation of the cytokine system during epileptic activity was reported by a number of studies. Seizures induced by focal application of kainate have been shown to cause microglial activation and an increase in interleukin-1β (IL-1β) production 24 h after kainate injection [4]. An increase in IL-1β expression has been found in rats between 12 and 24 h after lithium-pilocarpine seizures in areas most prone to neurodegeneration [5]. Activation of the IL-1β system in glia and neurons has been found in hippocampal samples from both animals and patients with temporal lobe epilepsy associated with hippocampal sclerosis [2,6]. An increase in IL-1β immunoreactivity was observed in the cortex and hippocampus of rats with spontaneous seizures 4 months after status epilepticus [2]. In clinical studies, an increase in the content of proinflammatory cytokines IL-1β, interleukin-6 (IL-6), and tumor necrosis factor-α (TNF-α) was found in the cerebrospinal fluid of patients with epilepsy after an epileptic seizure [7].

The endocannabinoid system is a primary regulator of synaptic transmission [8]. Neurons predominantly contain endocannabinoid CB1 receptors, which are the most abundantly expressed metabotropic receptors in the brain [9]. The expression of CB1 receptors has been found on presynaptic membranes in many brain structures, especially in the neocortex, hippocampus, basal ganglia, hypothalamus, and cerebellum [10]. Endocannabinoid CB1 receptors are located at the presynaptic membranes and carry out “on-demand” retrograde inhibition of neurotransmitter release [11]. Activation of the endocannabinoid system underlies depolarization-induced inhibition of synaptic transmission [12,13], so it plays an important role in maintaining the balance of inhibition and excitation, and hence in the generation of seizure activity and protection against excitotoxicity. Many studies have shown that endocannabinoid receptors play an important role in the termination of seizure activity. The activation of CB1 receptors has an anticonvulsive effect [14,15,16,17]; endocannabinoid antagonists facilitate seizure activity [18,19]. It was shown that endocannabinoid receptor agonists have a neuroprotective effect in vivo and in vitro [20,21,22].

In this study, we investigated the gene expression of proinflammatory cytokines IL-1β (*Il1b*) and IL-6 (*Il6*), and anti-inflammatory fractalkine (*Cx3cl1*) in the hippocampus, entorhinal cortex, and neocortex of rats at different time points after lithium-pilocarpine status epilepticus (SE). Our aim was to study how changes in cytokine expression are related to neuronal death in the hippocampus and evaluate the effect of modulation of the endocannabinoid system on cytokine expression and neuronal death at different time points after SE.

## 2. Results

### 2.1. Gene Expression in the Rat Brain in the Acute Period after SE

#### 2.1.1. Hippocampus

**Figure 1:**
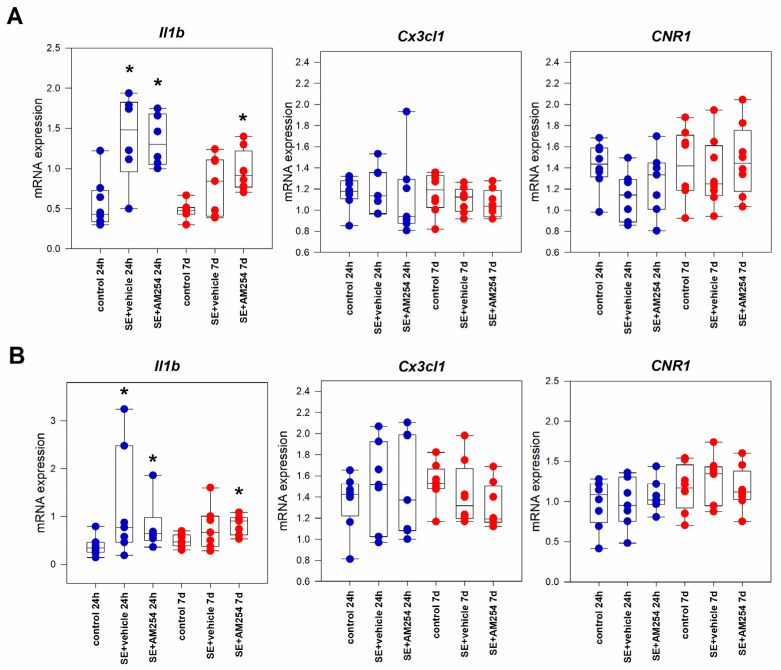
*Il1b*, *Cx3cl1*, and *CNR1* mRNA expression in the dorsal (**A**) and ventral (**B**) hippocampus 24 h and 7 days after SE in rats with no SE (control), vehicle-treated treated rats (SE + vehicle), and AM251-treated rats (SE + AM251). Data are represented as medians with interquartile ranges; dots represent individual values. * statistically significant differences at *p* < 0.05 in comparison with the corresponding control group, Kruskal–Wallis test.

The results of qPCR analysis of gene expression in the rat hippocampus showed a significant increase in the proinflammatory cytokine interleukin-1β (*Il1b*) gene expression in the dorsal hippocampus 24 h after lithium-pilocarpine status epilepticus (SE) in comparison with the control group (*p* = 0.0036, Kruskal–Wallis test, *p* = 0.008, multiple comparisons post hoc). This increase appeared to be less prominent 7 days after SE, but it was still significant in comparison with the control group (*p* = 0.017 and *p* = 0.031, Kruskal–Wallis test, with *p* = 0.017 and *p* = 0.029, multiple comparisons post hoc, in the dorsal and ventral hippocampus respectively) (Figure 1). The expression of anti-inflammatory chemokine fractalkine (*Cx3cl1*) did not change either 24 h or 7 days after SE. No statistically significant changes in the expression of CB1 receptors (*CNR1*) were found in the hippocampus 24 h and 7 days after SE. Administration of CB1 receptor antagonist AM251 did not significantly change the patterns of expression of the studied cytokines, including *CNR1* expression (Figure 1).

#### 2.1.2. Entorhinal Cortex

**Figure 2:**
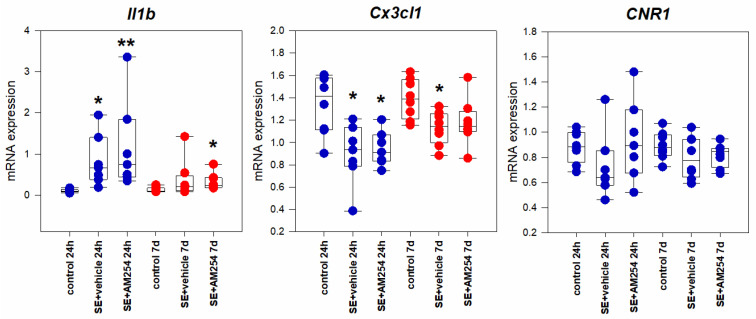
*Il1b*, *Cx3cl1*, and *CNR1* expression in the entorhinal cortex 24 h and 7 days after SE in rats with no SE (control), vehicle-treated treated rats (SE + vehicle), and AM251-treated rats (SE + AM251). Data are represented as medians with interquartile ranges; dots represent individual values for each rat. * statistically significant differences at *p* < 0.05; ** statistically significant differences at *p* < 0.005, in comparison with the corresponding control group, Kruskal–Wallis test.

The *Il1b* expression in the entorhinal cortex changed in a similar way as in the hippocampus: at 24 h, a significant increase in the expression was observed (*p* = 0.0006, Kruskal–Wallis test, *p* = 0.001, multiple comparisons post hoc), that was less prominent 7 days after SE (Figure 2). However, in contrast to the hippocampus, a significant decrease in the *Cx3cl1* expression was found 24 h (*p* = 0.016, Kruskal–Wallis test, *p* = 0.045, multiple comparisons post hoc) and 7 days (*p* = 0.030, Kruskal–Wallis test, *p* = 0.044, multiple comparisons post hoc) after SE (Figure 2). The expression of CB1 receptors did not change; administration of AM251 did not have any significant effect on the expression of the studied genes.

#### 2.1.3. Neocortex

**Figure 3:**
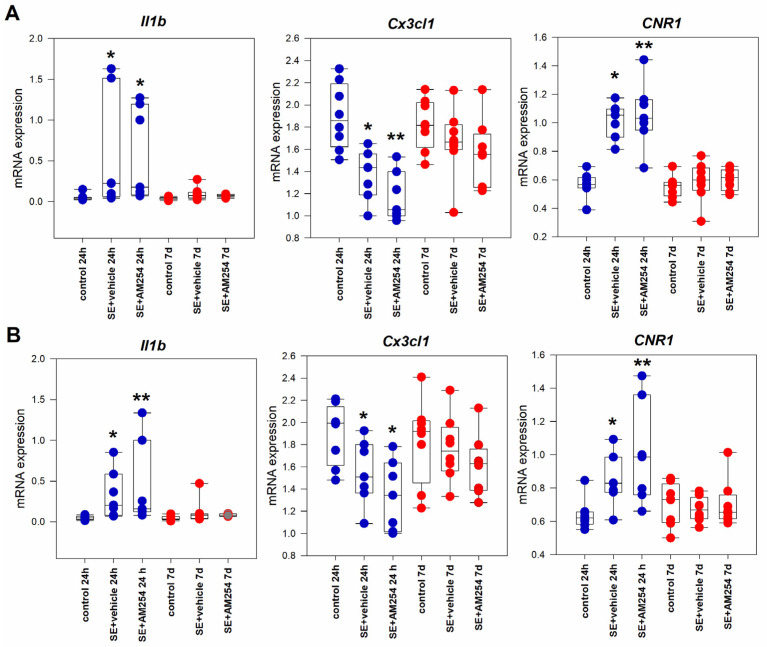
*Il1b*, *Cx3cl1*, and *CNR1* mRNA expression in the somatosensory (**A**) and frontal (**B**) cortexes 24 h and 7 days after SE in rats with no SE (control), vehicle-treated treated rats (SE + vehicle), and AM251-treated rats (SE + AM251). Data are presented as medians with interquartile ranges; dots represent individual values for each rat. * statistically significant differences at *p* < 0.05; ** statistically significant differences at *p* < 0.005, in comparison with the corresponding control group, Kruskal–Wallis test.

The expression of *Il1b* significantly increased both in the somatosensory (*p* = 0.011, Kruskal–Wallis test, *p* = 0.037, multiple comparisons post hoc) and frontal (*p* = 0.002, Kruskal–Wallis test, *p* = 0.010, multiple comparisons post hoc) cortexes 24 h after SE. The *Cx3cl1* expression, on the contrary, significantly decreased in the somatosensory and frontal cortex 24 h after the seizures (*p* = 0.001 and *p* = 0.013, Kruskal–Wallis test, *p* = 0.036 and *p* = 0.012, multiple comparisons post hoc, in the somatosensory and frontal cortexes, respectively). The *CNR1* expression in the somatosensory (*p* = 0.0008, Kruskal–Wallis test, *p* = 0.005, multiple comparisons post hoc) and frontal (*p* = 0.008, Kruskal–Wallis test, *p* = 0.009, multiple comparisons post hoc) cortexes changed in a similar way. The increase in *Il1b* and *CNR1* expression returned to the control levels 7 days after SE in the somatosensory and frontal cortex (Figure 3A,B). The blocking of CB1 receptors with AM251 did not have any significant effect on the expression of either cytokines *Il1b* and *Cx3cl1* or endocannabinoid receptors *CNR1* in the acute and latent periods after SE.

### 2.2. Neurodegeneration in the Acute and Latent Period after SE

**Figure 4:**
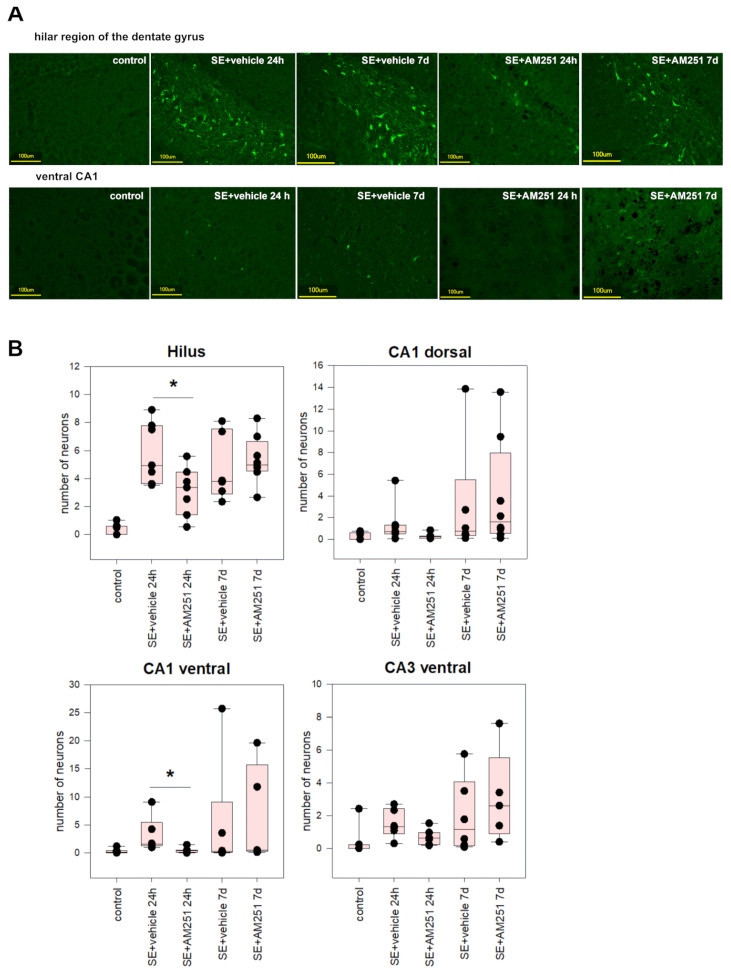
(**A**) Fluorojade C staining in the hilar region of the dentate gyrus (upper row) and CA1 of the ventral hippocampus (bottom row) in rats without SE (control), vehicle-treated treated rats (SE + vehicle), and AM251-treated rats (SE + AM251) 24 h and 7 days after SE; (**B**) the number of fluorescent neurons per field of view in rats with no SE (control), vehicle-treated treated rats (SE + vehicle), and AM251-treated rats (SE + AM251) 24 h and 7 days after SE. Data are presented as medians with interquartile ranges; dots represent individual values for each rat. *—significant differences at *p* < 0.05, Kruskal–Wallis test.

The fluorojade C staining of rat brain slices showed the presence of fluorescent cells in various structures with prominent staining in the CA1 and CA3 hippocampal fields and the dentate gyrus. In the acute period after SE (24 h), fluorescence was detected predominantly in the hilar region of the dentate gyrus and the ventral hippocampus (Figure 4A). Seven days after SE, staining in the CA1 region appeared to be more prominent. In the control brains, only single fluorescent cells were detected episodically.

#### 2.2.1. Neurodegeneration in the Dorsal and Ventral Hippocampus and the Effect of AM251

Degenerating cells in the CA1 of the hippocampus were detected as early as 24 h after SE and were still present a week after the initial insult. Fluorescent cell counts in the dorsal CA1 of vehicle-treated rats were 1.40 ± 0.69 (*n* = 7) 24 h after SE and 3.10 ± 2.18 (*n* = 6) 7 days after SE. In the AM251-treated rats, the number of fluorescent cells in CA1 was 0.29 ± 0.10 (*n* = 7) and 3.91 ± 1.75 (*n* = 8) 24 h and 7 days after SE, respectively. A comparison between the AM251-treated rats with the vehicle-treated group showed a decrease in fluorescent cells number 24 h after SE, but it was not statistically significant (Kruskal–Wallis test, Mann–Whitney U-test, *p* = 0.064). A week after SE, the level of neurodegeneration was the same in both groups (Figure 4B).

In the dentate gyrus, quite prominent neurodegeneration was observed 24 h after SE: the number of fluorescent cells was 5.83 ± 0.83 (*n* = 7). This remained at the same level: 4.75 ± 0.97 (*n* = 6) 7 days after SE. In AM251-treated rats, these numbers were 3.09 ± 0.66 (*n* = 7) and 5.25 ± 0.60 (*n* = 8), respectively. The number of fluorescent cells was reduced in the dentate AM251-treated rats in comparison with the vehicle-treated rats 24 h after SE (Kruskal–Wallis test, *p* = 0.035). This decrease was absent 7 days after SE (Figure 4B).

Neurodegeneration in the ventral hippocampus also was detected 24 h after SE and remained at the same level 7 days after SE. The number of fluorescent cells in the ventral CA1 of vehicle-treated rats was 3.14 ± 1.19 (*n* = 6) and 5.01 ± 4.18 (*n* = 6) 24 h and 7 days after SE respectively. In AM251-treated rats, the fluorescent neuron counts were 0.47 ± 0.18 (*n* = 7) and 6.50 ± 3.14 (*n* = 5) respectively. Administration of AM251 significantly reduced the number of fluorescent cells in the ventral CA1 24 h after SE (Kruskal–Wallis test, *p* = 0.008), but did not affect neurodegeneration 7 days after SE (Figure 4B).

Therefore, inhibiting CB1 receptors with AM251 4 h after SE reduced the number of neurodegenerating neurons in the acute period after SE, however, this effect was not sustainable and did not prevent further neurodegeneration during the latent period after SE.

#### 2.2.2. Effect of WIN-55,212-2 on Neurodegeneration in the Acute and Latent Period after SE

To compare the effect of inhibition of CB1 receptors by AM251 with the effect of activation of the endocannabinoid system on the neurodegeneration in the hippocampus in the acute and latent period after SE, the effect of a potent endocannabinoid agonist WIN-55,212-2 was studied.

**Figure 5:**
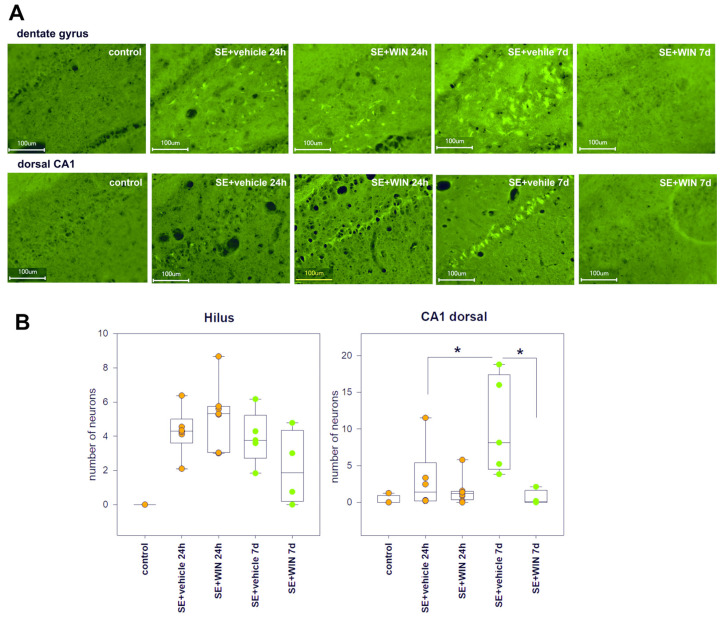
(**A**) Fluorojade C staining in the hilar region of the dentate gyrus (upper row) and dorsal CA1 (bottom row) in rats without SE (control), vehicle-treated treated rats (SE + vehicle), and WIN-55,212-2-treated rats (SE + WIN) 24 h and 7 days after SE; (**B**) The number of fluorescent cells per field of view in the dentate gyrus and CA1 of the dorsal hippocampus in rats with no SE (control), vehicle-treated treated rats (SE + vehicle), and WIN-55,212-2-treated rats (SE + WIN) 24 h and 7 days after SE. Data are presented as medians with interquartile ranges; dots represent individual values for each rat. *—significant differences at *p* < 0.05, Kruskal–Wallis test, multiple comparisons post hoc.

The mean number of fluorescent cells in the dorsal CA1 of vehicle-treated rats was 3.00 ± 1.79 cells per field of view 24 h after SE (*n* = 6) and 10.39 ± 2.97 cells per field of view 7 days after SE (*n* = 6). In WIN-treated rats, the number of fluorescent cells was 1.52 ± 0.64 (*n* = 8) and 0.57 ± 0.51 (*n* = 4) 24 h and 7 days after SE respectively. The number of fluorescent cells in the CA1 of the dorsal hippocampus tended to increase 7 days after SE in comparison with the number of fluorescent cells 24 h after SE, but at the same time, it was significantly reduced in the WIN-treated rats in this time period (*p* = 0.003, Kruskal–Wallis test, *p* = 0.032, multiple comparisons post hoc) (Figure 5).

In the hilar region of the dentate gyrus, the number of fluorescent cells in the vehicle-treated rats was 7.84 ± 1.79 cells per field of view (FOV) 24 h after SE (*n* = 6) and 9.75 ± 2.47 cells per FOV 7 days after SE (*n* = 5). In WIN-treated rats, these numbers were 5.80 ± 2.05 (*n* = 8) and 5.19 ± 2.93 (*n* = 4) 24 h and 7 days after SE, respectively. Fluorescent cell counts were lower in WIN-treated rats, but this difference was not statistically significant (Figure 5).

In the ventral hippocampus, the level of fluorescent staining in the control group and the rats after SE was found to be practically the same; no significant differences were found in the number of fluorescent cells in the vehicle- and WIN-treated rats 24 h and 7 days after SE.

Therefore, the comparison of the number of fluorescent cells in the hippocampus of rats treated with WIN-55,212-2 and rats treated with vehicle 4 h after SE showed that WIN-55,212-2 treatment significantly reduced the number of fluorescent cells in the CA1 7 days after SE; in the dentate gyrus, this decrease was not statistically significant.

### 2.3. Gene Expression and Neurodegeneration in the Rat Brain in the Chronic Period after SE

Video monitoring of rats 5 months after SE showed the development of spontaneous seizures in both vehicle-treated and AM251-treated rats. In the vehicle-treated group, three of eight rats that were video-monitored and included in the qPCR analysis had spontaneous generalized tonic-clonic seizures; in the AM251-treated group, six of seven rats had spontaneous seizures. The average number of seizures in vehicle-treated and AM251-treated rats was 0.48 ± 0.27 and 1.15 ± 0.4 per day, respectively. It seemed that AM251-treated rats tended to develop spontaneous seizures more frequently, but this difference was not statistically significant.

**Figure 6:**
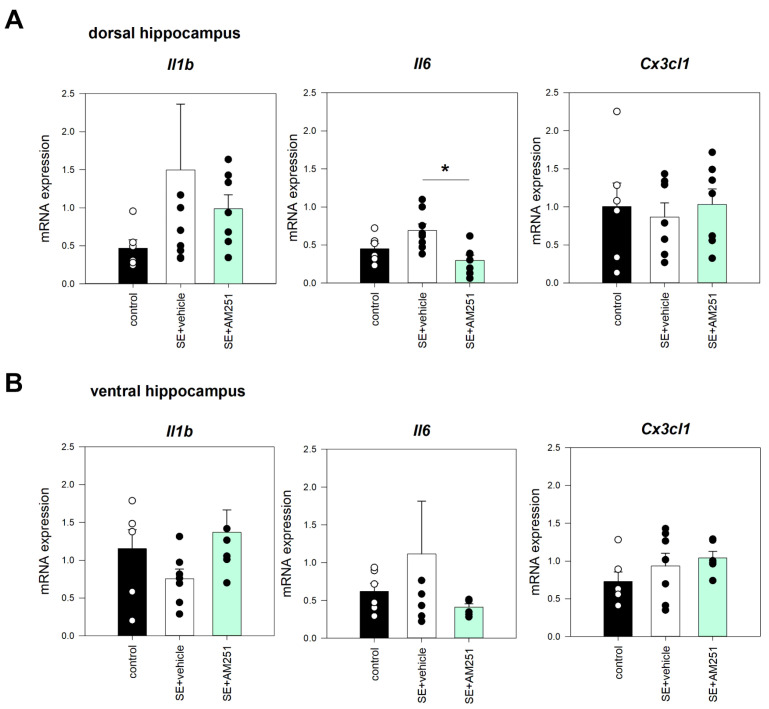
*Il1b*, *Cx3cl1*, and *Il6* mRNA expression in the dorsal (**A**) and ventral (**B**) hippocampus 5 months after SE in rats with no SE (control), vehicle-treated treated rats (SE + vehicle), and AM251-treated rats (SE + AM251). Data are presented as mean ± SEM; dots represent individual values for each rat. *—statistically significant differences at *p* < 0.05, ANOVA, post hoc.

In the chronic period after SE, when a significant decrease in neuronal counts occurred, no significant changes in the expression of *Il1b* and *Cx3cl1* were found in the dorsal and ventral hippocampus (Figure 6A,B). We additionally investigated the expression of *Il6* in the hippocampus and cortex. Inhibition of CB1 receptors with a selective CB1 antagonist AM251 early after SE led to a decrease in the Il6 expression in the dorsal hippocampus of AM-251 treated rats in comparison with vehicle-treated rats 5 months after SE (*p* = 0.014, F = 7.576, ANOVA; *p* = 0.048, Tukey post hoc) (Figure 6A). No significant changes were found in the cortex of rats after SE compared to the control rats. Changes in the *Il1b* and *Cx3cl1* expression were not significant in the dorsal and ventral hippocampus; inhibition of CB1 receptors early after SE did not have an effect on the expression of these cytokines in the chronic period after SE.

In the chronic period after SE, we evaluated neurodegeneration in the hippocampus based on the number of cresyl-stained neurons in CA1, CA3, and the dentate gyrus 5 months after SE.

A significant reduction in cell counts was found in the dentate gyrus of rats survived after SE in comparison to the control rats (ANOVA, *p* = 0.003, F = 4.024, Tukey post hoc test); administration of AM251 did not have an effect on this reduction. In CA1 and CA3, there was no significant decrease in cell numbers in rats after SE compared to the control rats.

**Figure 7:**
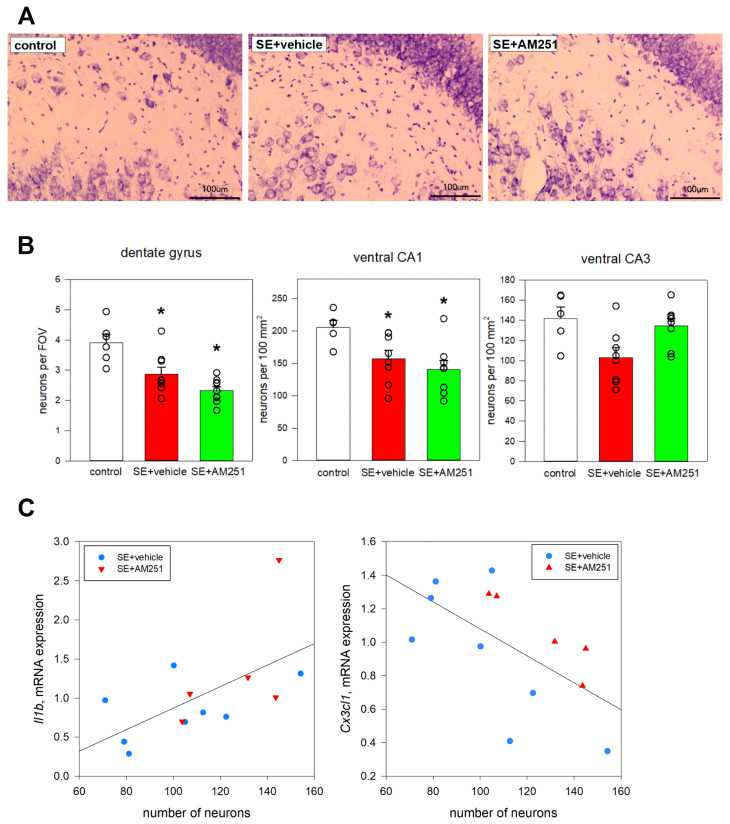
(**A**) neurodegeneration in the dentate gyrus of vehicle-treated and AM251-treated rats 5 months after SE. The control rats without SE had visibly more neurons, especially in the hilus of the dentate gyrus; (**B**) the neuronal count in the hippocampus of rats with no SE (control), vehicle-treated treated rats (SE + vehicle), and AM251-treated rats (SE + AM251) 24 h and 7 days after SE. Data are presented as mean ± SEM; dots represent individual values for each rat. *—significant differences at *p* < 0.05, ANOVA; (**C**) correlations between the number of neurons in the ventral hippocampus (X-axis) and the expression of *Il1b* (Spearman correlation 0.59, *p* < 0.05) and *Cx3cl1* (Spearman correlation −0.61, *p* < 0.05) (Y-axis) 5 months after SE.

In the ventral hippocampus, a significant decrease in neuronal counts was found in CA1 (*p* = 0.017, F = 3.409, ANOVA, Tukey post hoc test); AM251 did not affect the decrease in neuronal counts (Figure 7B).

There was no difference in neuronal counts between rats with chronic spontaneous seizures and rats with no spontaneous seizures.

Even though no statistically significant changes were found in the expression of the studied genes in rats survived after SE, there was individual variability, and some animals had elevated levels of *Il1b* expression. We decided to investigate if gene expression depended on neurodegeneration. There was a correlation between the number of neurons and the expression of Il1b (Spearman correlation 0.59, *p* < 0.05) and *Cx3cl1* (Spearman correlation −0.61, *p* < 0.05) in the ventral hippocampus (Figure 7C). Interestingly, higher *Il1b* levels were found in rats with higher neuronal counts in the ventral hippocampus; on the contrary, higher levels of fractalkine were associated with more prominent neuronal loss.

## 3. Discussion

In our study, we investigated the changes in the mRNA expression of pro- and anti-inflammatory cytokines interleukin-1β (IL-1β, *Il1b*), interleukin-6 (IL-6, *Il6*), and fractalkine (*Cx3cl1*) at different time points after prolonged severe seizures: during the acute period (24 h), latent period (7 days), and chronic period (5 months). We also studied the effect of modulation of the endocannabinoid system on seizure-induced neurodegeneration and its relationship with cytokine and endocannabinoid receptor CB1 (*CNR1*) expression.

Our main findings include: (1) an increase in mRNA level of *Il1b* and *CNR1* and a decrease in *Cx3cl1* expression were found in the neocortex, while only *Il1b* expression changed in the hippocampus in the studied time periods after SE. The most persistent decrease in *Cx3cl1* expression was found in the entorhinal cortex; (2) inhibition of CB1 receptors early after SE had a transient neuroprotective effect that did not appear in the chronic period and did not prevent the development of spontaneous seizures after SE; (3) inhibition of CB1 receptors prevented an increase in IL6 expression in the chronic period after SE; (4) higher *Cx3cl1* levels were found in rats with more prominent hippocampal neurodegeneration in the chronic period after SE.

It is well established that the serum and cerebrospinal fluid levels of proinflammatory cytokines can be elevated in epilepsy patients [23,24]. The increase in cytokine levels was observed both postictally [7] and interictally [25]. This upregulation of proinflammatory cytokines evidently causes rapid and massive activation of inflammatory response in the brain, which has been reported by many studies [26]. In the experimental seizures, protein and gene upregulation of IL-1β and IL-6 was found a few hours after SE onset and was still present days after SE or kainate application [4,27,28]. Therefore, the increase in *Il1b* expression in the rat hippocampus and cortex 24 h after SE in the present study is consistent with the results obtained in other studies carried out on pilocarpine and other models. In our study, the Il1b in the hippocampus decreased 7 days after SE but was still significantly higher than in the control group. The same dynamics were observed in the adjacent cortex; but in the neocortex, elevated *Il1b* expression evidently decreased faster than in the hippocampus and the adjacent cortex.

A rapid transient increase in IL-6 expression was demonstrated in the rat hippocampus in the acute period after SE and in rats with spontaneous seizures in the chronic period after SE [28,29]. In our study, the *Il6* expression in the chronic period after SE, when spontaneous seizures have developed, tended to increase in the hippocampus but did not change in the adjacent cortex and the neocortex. In CSF and serum of epilepsy patients, IL-6 levels were shown to correlate with the severity of seizures [30,31], but we have not found any relationship between the presence of spontaneous seizures and *Il6* expression in the rat brain in the chronic period after SE.

In contrast to proinflammatory IL-1β and IL-6, fractalkine is believed to reduce the proinflammatory response in the brain [32]. Long-term upregulation of fractalkine expression was reported in the hippocampus and adjacent cortex of rats after lithium-pilocarpine SE, as well as in the temporal cortex and CSF of patients with temporal lobe epilepsy [33]; however, in that case, an increase in protein concentrations was found by IHC and ELISA, and gene expression was not studied. Another study on the pilocarpine model showed an increase in fractalkine immunoreactivity 1–3 h after SE, and a decrease 3 days after SE [34]. In the present study, we did not find changes in the *Cx3cl1* mRNA expression in the hippocampus, probably because we selected different time points, however, we did find a transient decrease in the *Cx3cl1* expression in the neocortex 24 h after SE and more persistent *Cx3cl1* downregulation in the entorhinal cortex 24 h and 7 days after SE. Downregulation of mRNA expression of *Cx3cl1* after SE could be one of the underlying mechanisms of seizure-induced neuroinflammation and neurodegeneration. We have not found any significant correlations of *Cx3cl1* mRNA expression with neurodegeneration in the hippocampus early after SE, however, in the chronic period, higher levels of *Cx3cl1* mRNA expression were associated with more prominent neuronal loss.

Selective inhibition of CB1 receptors 4 h after SE did not affect the levels of *Il1b* and *Cx3cl1* expression. Inhibition of CB1 receptors early after SE suppressed the increase in the expression of *Il6* in the hippocampus in the chronic period. There are reports that IL-6, while promoting inflammatory response, can have a neuroprotective effect via the JAK/STAT3 and RAS/MAPK pathways [35,36,37], and suppression of *Il6* expression could aggravate neurodegeneration [37]. Other studies showed that in epilepsy models, *Il6* upregulation could aggravate neuronal damage [38]. In our study, *Il6* expression did not correlate with neuronal loss in the hippocampus in the chronic period after SE, so the effect of modulation of the endocannabinoid system on *Il6* expression likely was not associated with the processes of neurodegeneration.

It is interesting that *Il1b* and *Cx3cl1* expression, which was not affected by modulation of the endocannabinoid system early after SE, on the contrary, correlated with neuronal loss in the ventral hippocampus. Elevated levels of *Il1b* corresponded to less severe neuronal loss, which could be due to the larger number of *Il1b*-expressing cells in comparison to the brains with more prominent neuronal loss. Elevated levels of *Cx3cl1* expression corresponded to more severe neurodegeneration in the hippocampus. Multiple studies have shown the neuroprotective effects of fractalkine [39,40,41]. However, it was reported that in the pilocarpine SE model, fractalkine could promote neurodegeneration via microglial/monocyte activation rather than neuroprotection via BDNF release [34]. Our results suggest that fractalkine could have a neurodegenerative effect in the chronic period after SE when spontaneous seizures have already developed. At the same time, there was no indication that *Cx3cl1* expression depended on the presence of spontaneous seizures, so there is possible that the initial insult such as SE could lead to long-term disruption of *Cx3cl1* expression.

Finally, even though inhibition of CB1 receptors by AM251 early after SE had no effect on either cytokine or CB1 receptor expression, it reduced the number of degenerating neurons in the hippocampus of rats 24 h after SE. However, this effect was transient and was not observed in the latent and chronic periods after SE. On the contrary, the effect of the administration of endocannabinoid agonist WIN 55,212-2 was observed in the latent period after SE. According to our previous findings, WIN 55,212-2 decreases neuronal loss in the hippocampus in the chronic period after SE, i.e., it had a long-term neuroprotective effect [42], unlike the CB1 antagonist. The proconvulsive effect of CB1 antagonists [15,18] and the anticonvulsive and neuroprotective effects of CB1 agonists [14,42,43,44] have been reported in various models of seizure activity. In the present study, both activation and inhibition of endocannabinoid receptors had a neuroprotective effect. It is well-known that CB1 receptors are abundantly present on presynaptic membranes of both GABAergic [45] and glutamatergic neurons [11], and it was shown that a population of CB1 receptors located on the glutamatergic neurons is responsible for neuroprotection [46], and the protective effect of endocannabinoid agonists is carried out via blockage of glutamatergic neurotransmission by activation of presynaptic CB1. However, we should note that WIN 55,212-2 is a nonselective agonist and can also activate CB2 receptors, thus suppressing neuroinflammation, which could contribute toward its lasting neuroprotective effect. At the same time, blockage of CB1 receptors on GABAergic neurons could prevent the suppression of GABA-mediated inhibition and thus reduce excitotoxic neuronal damage. Interestingly, there are reports that the prevention of the activation of endocannabinoid signaling induced by seizures early after SE can attenuate subsequent epileptogenesis [47,48], probably due to the block of suppression of GABA-ergic transmission, which led to a decrease in hippocampal hyperexcitability. In our study, no changes in the incidence or frequency of spontaneous seizures and no changes in neurodegeneration in the chronic period after SE were found despite the transient suppression of neurodegeneration by AM251 early after SE. However, the suppression of epileptogenesis was observed in the developing brain [47,49] and after the prolonged administration of a CB1 antagonist [48], while single administration of a CB1 antagonist in the adult brain did not have this effect. Moreover, the expression of CB1 receptors in the early and latent period also was not affected by CB1 inhibition, which could indicate that it was insufficient for modifying the long-term outcome of SE.

## 4. Materials and Methods

### 4.1. Animals

The study was carried out on adult male Wistar rats weighing 200–250 g at the beginning of the experiment. Rats were purchased from the Scientific Center for Biomedical Technologies of the Federal Medical and Biological Agency, Russia. The animals were kept under standard vivarium conditions with 24 h light/dark cycle and free access to food and water. All experimental procedures were conducted in accordance with Directive 2010/63/EU for animal experiments and local regulations. The study protocol was approved by the Ethics Committee of the Institute of Higher Nervous Activity and Neurophysiology of the Russian Academy of Sciences.

### 4.2. Drugs

The study was carried out on the lithium-pilocarpine model of status epilepticus (SE). Rats were treated with 127 mg/kg lithium chloride (Acros Organics, Trenton, NJ, USA) 24 h before the induction of seizures. SE was induced by the administration of 25 mg/kg pilocarpine hydrochloride (Sigma, St. Louis, MO, USA). For the long-term survival of animals, seizures were stopped by administration of 0.6 mL/kg paraldehyde (Sigma, St. Louis, MO, USA) dissolved in saline. A reverse agonist (inhibitor) AM251 (Santa-Cruz Biotechnology, Dallas, TX, USA) or a potent agonist of CB1 and CB2 receptors WIN-55,212-2 (Sigma, St. Louis, MO, USA) was administered 4 h after the termination of SE. Lithium chloride and pilocarpine were freshly dissolved in 0.9% saline. AM251 and WIN-55,212-2 were dissolved in 5% DMSO (Sigma, St. Louis, MO, USA) and 1% Tween-80 (Panreac, Barcelona, Spain). All drugs were injected intraperitoneally.

### 4.3. Experimental Design

Samples of brain tissues of rats survived after SE were gathered 24 h, 7 days, and 5 months after lithium-pilocarpine SE was induced. In 4 h after SE arrest, CB1 receptors antagonist AM251 or vehicle were injected intraperitoneally. The control rats received saline instead of pilocarpine and did not develop seizures. The following experimental groups were included in the study: control 24 h; control 7 days; control 5 months; SE + vehicle 24 h, 7 days, and 5 months; SE + AM251 24 h, 7 days, and 5 months after SE. From each animal, samples from one hemisphere were gathered for qPCR, the other hemisphere was fixated in 4% paraformaldehyde for the histologic analysis.

In addition, the effect of the nonselective endocannabinoid agonist WIN-55,212-2 on neurodegeneration was studied. WIN-55,212-2 was administered 4 h after SE termination; control rats received vehicle. The following experimental groups were included in the study: SE + vehicle 24 h, SE + vehicle 7 days, SE + WIN 24 h, SE + WIN 7 days; the control group included samples gathered 24 h and 7 days after SE. Brains from these animals were gathered only for histologic analysis.

### 4.4. Video Monitoring

Five months after SE, rats were video-monitored to detect motor seizures. Video was recorded using a 4-channel video-monitoring system (Best DVR, Moscow, Russia) and digital mini video cameras with a 6 mm lens placed above the cages with animals. Video was recorded at 25 frames per second and a resolution of 352 × 288 pixels. During the dark hours, recordings were carried out under dim red light. Animals were video-monitored for 7 days. Videos were analyzed manually; clonic and tonic-clonic seizures were detected visually and scored according to the Racine scale [50].

### 4.5. Histological Procedures

Brains were removed, fixated in 4% paraformaldehyde, cryoprotected in 20% sucrose, frozen, and stored at −20 °C. Coronal slices 20 µm in size were obtained, mounted on slides, and dried. Each second and third slice was collected for further staining with Fluorojade C (FJC, Merck Millipore, Darmstadt, Germany) or cresyl violet acetate (Acros Organics, NJ, USA).

For performing neuron counts, three consecutive slices were picked from the stack of hippocampal slices corresponding to 3.3–3.6 mm posterior to the bregma for the dorsal hippocampus and 4.5–4.8 mm posterior to the bregma for the ventral hippocampus.

FJC staining was evaluated in the rat hippocampus using a BZ-9000 fluorescence microscope (Keyence, Osaka, Japan) 24 h and 7 days after SE. Exposure time was adjusted for each image to clearly show the fluorescent signals. After acquiring the images, the brightness and contrast were adjusted in some images using ImageJ. Fluorescent cells were counted using an ImageJ cell counter in a 130 × 170 µm (0.022 mm^2^) field of view (FOV); the average numbers per FOV from three consecutive slices with four FOVs each were calculated for every brain. Fluorescent neurons were counted in the CA1 and CA3 fields of the dorsal and ventral hippocampus and the hilar region of the dentate gyrus of the dorsal hippocampus. Cell counting was performed manually using an ImageJ cell counter by an observer blinded to the grouping of animals.

Neuronal counts in the cresyl-stained brain slices were evaluated in the CA1 and CA3 fields of the dorsal and ventral hippocampus and the hilus of the dentate gyrus of rats in the chronic period (5 months) after SE. For this purpose, high-resolution images were obtained using a BZ-9000 fluorescence microscope (Keyence, Osaka, Japan). Cell counts were assessed in three 0.023 mm^2^ FOVs for three consecutive slices for CA1 and CA3 fields, and in seven 0.011 mm^2^ fields of view on three consecutive slices for the hilus of the dentate gyrus; neuronal counts in the CA1 and CA3 fields were presented as a number of neurons per 0.1 mm^2^ and as a number of neurons per FOV in the hilus of each rat. Cell counting was performed manually using an ImageJ cell counter by an observer blinded to the grouping of brains. Only neurons with a cell body larger than 0.01 mm were counted.

### 4.6. qPCR Analysis

To carry out qPCR, the following brain structures were extracted and immediately frozen in liquid nitrogen: dorsal and ventral hippocampus, entorhinal cortex, somatosensory cortex, and frontal cortex.

Total RNA was isolated using ExtractRNA reagent (Evrogen, Moscow, Russia). To remove traces of genomic DNA, RNA samples were treated with DNase I (Thermo Scientific, Waltham, MI, USA). Reverse transcription was performed using the MMLV RT kit reagent kit (Evrogen, Moscow, Russia) using murine RNase Inhibitor (New England Biolabs, Ipswich, MA, USA). An equimolar mixture of random decaprimer (Evrogen, Moscow, Russia) and Oligo(dT)15 primer (Evrogen, Moscow, Russia) was used; the concentration of each primer in the reaction was 1 µM. After reverse transcription, the reaction mixture was diluted eightfold with deionized water.

The mRNA expression of the following genes was analyzed: *Cx3cl1*—chemokine (C-X3-C motif) ligand 1 (CX3CL1, fractalkine), *Il1b*—interleukin-1β, *IL6*—interleukin-6, *CNR1*—endocannabinoid receptor CB1.

Relative quantities of mRNAs for the genes of interest were measured in a Bio-Rad CFX-384 real-time PCR station using a qPCRmix-HS SYBR + LowROX PCR mix for PCR (Evrogen, Moscow, Russia) according to the manufacturer’s recommendations. Relative quantities of mRNAs for genes expressed in the different parts of the neocortex and hippocampus were normalized to the geometric mean of the mRNA expression levels for the *Ywhaz*, *Osbp*, and *Hprt1* genes. Relative quantities of mRNA in the dura mater and meninges were normalized to the geometric mean of the mRNA expression levels for the *Ywhaz* and *Hprt1* genes. To assess the quality of the DNase treatment for all the samples and genes, we ran a negative control with the product of DNase I treatment. Gene expression was analyzed by the E^−ΔΔCt^ method. The sequences of used primers are shown in Table 1.

### 4.7. Statistical Analysis

Statistical analysis was carried out using Statistica software 12.0 (StatSoft, Tulsa, OK, USA). All data samples were tested for the normality using Shapiro–Wilk test. If the data distribution was normal, the difference between samples was analyzed using ANOVA with Tukey’s post hoc test. Data with distributions that did not pass the normality Shapiro–Wilk test were evaluated using the nonparametric Kruskal–Wallis test with post hoc multiple comparisons of mean ranks of studied groups. Correlation analysis was carried out using the nonparametric Spearman correlation test.

## Figures and Tables

**Table 1:** Sequences of primers and annealing temperatures used for each primer pair (5′ > 3′).

Gene	T_annealing_, °C	Forward	Reverse
*osbp*	65	TCC GGG AGA CTT TAC CTT CAC TT	GTG TCA CCC TCT TAT CAA CCA CC
*Il1b*	61	TCT GTG ACT CGT GGG ATG AT	CAC TTG TTG GCT TAT GTT CTG TC
*cx3cl1*	61	ATC ACC ACC ATC ACC ACC AAC	GAG GAA CAC TTT AAA CCC TCA CAG A
*cnr1*	65	CGG CAT CTC TTT CTC AGT CA	CTG CGG TCA TCT TTT CTT GG
*ywhaz*	63	TTG AGC AGA AGA CGG AAG GT	GAA GCA TTG GGG ATC AAG AA
*hprt*	65	CGT CGT GAT TAG TGA TGA TGA AC	CAA GTC TTT CAG TCC TGT CCA TAA

## Data Availability

Data are contained within the article.
